# Prevalence and Related Factors of HIV Screening Adherence Among Pregnant Women in Primary and Secondary Healthcare Facilities: A Cross-Sectional Study

**DOI:** 10.3390/tropicalmed11100269

**Published:** 2026-09-28

**Authors:** Mina Benaddi, Abdelhafid Benksim, El Mostafa Atiki, Sanaa SABOUR ALAOUI

**Affiliations:** 1 Applied Biology for Health, Environment and Sustainable Development Team (BASED), Department of Biology and Geology, Polydisciplinary Faculty of Beni Mellal, Sultan Moulay Slimane University, Beni-Mellal 23000, Morocco; sanaa_ala@yahoo.fr; 2 Nursing Care Department, Higher Institute of Nursing Professions and Health Techniques (ISPITS-M), Regional Health Directorate, Marrakech 40000, Morocco; benksima@gmail.com; 3 Legal Studies Department, Higher Institute of Nursing Professions and Health Techniques (ISPITS-BM), Regional Health Directorate, Beni-Mellal 23000, Morocco; doctorat3@hotmail.com

**Keywords:** HIV screening, MTCT, pregnant women, screening adherence

## Abstract

Background: The mother-to-child transmission of HIV infection prevention is a cornerstone in the control of the epidemic. HIV screening in pregnant women and timely administration of antiretroviral treatment are key actions in this prevention. This study aimed to describe the HIV screening adherence among pregnant women and its related factors in Beni-Mellal city, Morocco. Materials and methods: A cross-sectional study was conducted on 250 pregnant women attending facilities offering prenatal care services in Beni-Mellal, Morocco, between March and June 2025. Participants were collected using a non-probabilistic sampling. The sociodemographic, economic and clinical characteristics were collected through a guided interview questionnaire. A non-probabilistic Qui-square test was used to determine factors associated with the HIV screening adherence. A binomial logistic regression model was applied to determine potential factors influencing this adherence. Statistical significance was set at *p* < 0.05. Results: The rate of women that took the HIV test was 43.2%. The data analysis revealed the factors to be positively associated with HIV screening adherence to be previous knowledge about the test (*p* = 0.001, and the healthcare team proposing the test (*p* = 0.001). Conclusions: The systematization of HIV testing offered during pregnancy should be reinforced and the fight against stigmatization should be strengthened in health strategies to improve HIV screening adherence and enhance the prevention of mother-to-child transmission.

## 1. Introduction

The transmission of the human immunodeficiency virus (HIV) from mother to child, without any intervention, ranges between 15 per cent and 45 per cent, and half of all infants infected with HIV are likely to die before their second birthday if they do not receive treatment [1]. Reducing HIV transmission from mother to child requires a range of interventions, beginning with voluntary and confidential counseling and testing for the virus, followed by lifelong antiretroviral treatment for pregnant women with HIV and the administration of antiretroviral drugs to their newborns as a preventive measure, as well as safe delivery practices, and retention of the mother and infant into long-term care and treatment, including maintaining suppressed viral load levels [2]. There have been gains in recent years to ensure that women who are not already on lifelong antiretroviral treatment before their current pregnancy are newly and timely initiated to prevent mother-to-child transmission.

In 2024, about 120,000 new HIV infections among children under five occurred, with 62% decline in comparison with 2010 [2]. In fact, this drastic decrease can be partly attributed to the launch in 2010 of the global plan towards the elimination of new HIV infections among children by 2015 and keeping their mothers alive (EMTCT), which was a global goal to guarantee access to quality life-saving HIV prevention and treatment services to all women, especially pregnant women, for themselves and their children [3]. The plan insists on the community’s responsibility to support pregnant women and their partners in accessing HIV testing and counseling services without stigmatization and discrimination, and it calls on the national and sub-national authorities to exert their concerted leadership to enable this to happen [3]. The elimination of new HIV infections among children is heavily dependent on the availability of key commodities, such as antiretroviral drugs and technologies used in rapid HIV tests, CD4 counts, and viral load tests, including for early infant diagnostics. In many countries, access to these commodities is limited, and supply chain management systems are overstretched and unable to meet demand. Current program approaches are insufficient to reach the goal of eliminating new HIV infections among children and keeping their mothers alive. HIV prevention and treatment services and their delivery systems have to be simplified, with care provision at the Primary Health Care level. This includes rapid HIV testing, point-of-care diagnostics (CD4 counts) of pregnant women living with HIV, and simple one-pill daily drug regimens that do not have to be switched between pregnancies and breastfeeding periods [4].

In Morocco, the total number of children living with HIV was estimated at less than 1000 at the end of 2019 [5]. Regarding the mother-to-child transmission (MTCT), fewer than 100 HIV infections occur each year [6]. To address this epidemiological situation, the Moroccan Health Ministry, with the support of its partners, has developed and implemented proactive national strategies to facilitate access to HIV prevention, screening, and care services for key populations and people living with HIV (PLHIV). Furthermore, the health department launched several actions to fight stigmatization and ensure respect for human rights and gender equality.

The 2023 National Strategic Plan (NSP) is consistent with the three 95-95-95 strategy, aiming to end the AIDS epidemic by 2030 [7]. The strengthening of HIV screening, to reduce the number of late diagnoses and missed opportunities, has made it possible to reach 388,141 people tested and counseled in more than 1600 health facilities offering the service, with an estimated 78% of PLHIV knowing their HIV status by the end of 2018, which is well below the first 95% [7].

HIV screening is a crucial public health measure that identifies infected individuals so that they can be offered ART and limit the transmission of the virus [4]. In 2023, 410 pregnant women were living with HIV, with a positivity rate of 0.22% nationally [8]. In Morocco, voluntary testing must be proposed to all patients with an STI or tuberculosis, all pregnant women, partners of PLHIV, and anyone belonging to the HIV key population. Indeed, early screening of pregnant women is recommended to prevent vertical transmission (from mother to child). A roadmap for the elimination of MTCT of HIV has been implemented. MTCT coverage for pregnant women living with HIV reached 71% in 2019 [9].

Pregnancy therefore provides an opportunity for HIV testing that should not be missed, both to improve early diagnosis figures in order to accelerate the achievement of the first 95% target with a view to achieving the goal of controlling the epidemic by 2030, and to reduce vertical transmission and limit the number of new infections among children [10]. Several studies were conducted to identify the determinants of pregnant women’s adherence to HIV screening during pregnancy. Those factors were essentially predisposing factors (age, educational level, and knowledge about HIV), enabling factors (family support, health workers’ support, and facilities characteristics), and reinforcing factors (perception of risk, and perception of benefit of screening) [11,12,13]. In the national territory, there is a lack of studies dedicated to the subject, and there are no papers, as far as we know, in the region of Beni-Mellal, discussing the HIV screening adherence among pregnant women. Thus, the current study was conducted to describe the rate of HIV testing among pregnant women in the province of Beni-Mellal and to identify the factors associated with this rate.

## 2. Materials and Methods

### 2.1. Study Design and Sitting

A quantitative cross-sectional study was conducted in order to describe the rate of HIV screening among pregnant women and its associated factors in Beni-Mellal city, Morocco.

The study was conducted at Oulad Hamdane Health Center, the Rmila Health Center, and the maternity of the Regional Hospital of Beni-Mellal. These three sites were chosen in order to combine the two levels of care (primary and secondary, curative and preventive). Indeed, the first and the second centers are both facilities offering primary health care services, including pregnancy and post-birth follow-up. Regarding the regional hospital maternity, it is a referent maternity for the region of Beni Mellal-khenifra, offering prenatal, delivery and postnatal health care services for the five provinces of the region.

### 2.2. Population, Sampling, and Sample Size

#### 2.2.1. Population

The target population for the study was pregnant women in the province of Beni-Mellal. The accessible population to the study was:

- Pregnant women recruited for antenatal care at health centers, notably the two centers mentioned above, chosen for their accessibility and the large number of pregnant women recruited in prenatal care services;
- Women admitted to the regional hospital maternity ward.

#### 2.2.2. Sampling and Sample Size

A non-probabilistic accidental sampling was used. The sampling procedure was motivated by the lack of a database of all pregnant women in the province and by matters of accessibility. The sample size was calculated based on the formula:

n = Z^2^·*p*·(1 − *p*)\e^2^

- **Z = 1.96 (95% confidence level);**
- ***p* = estimated proportion (0.05 if 5% of women are pregnant);**
- **e = margin of error (0.05): n = 1962·0.05·0.95\0.05^2^ ≈ 73**.

The obtained size was corrected as the population is a large one (proportion of pregnant women in the city: 40% sub-population; and a 2% pregnancy rate of all 209,594 accessible inhabitants in the province) [14]. A total number of 250 was obtained as a minimal representative sample of pregnant women in Beni-Mellal city with an error margin of 5.7% at the 95% confidence interval.

Then all pregnant women accessible during the data collection period were recruited and included till the calculated number was reached. All recruited pregnant women fully responded to our survey (100% response rate).

#### 2.2.3. Inclusion and Exclusion Criteria

- Inclusion criteria:

All pregnant women followed up at the selected health centers and the maternity of the regional hospital of Beni-Mellal able to understand and answer the questionnaire.

- Exclusion criteria:

All pregnant women presenting cognitive disorders or other disabilities preventing them from completing the questionnaire.

### 2.3. Data Collection Tools

Data was collected using a structured questionnaire developed based on a theoretical and empirical *PRECEDE-PROCEED model*, which is a validated framework assessing health-associated behavior’s adoption and change [15].

The questionnaire was tested on 5 pregnant women who were later excluded from the study.

The questionnaire was designed to collect pregnant women’s knowledge, perceptions, and practices regarding HIV testing during pregnancy. It includes 24 structured questions, divided into three main sections about predisposing, enabling and reinforcing factors, allowing for a systematic exploration of the cognitive, contextual, and social dimensions likely to influence the decision to undergo testing:

- Predisposing factors (age, education level, marital status, etc.);
- Enabling factors (access to information and HIV screening, health workers’ support, facilities accessibility);
- Reinforcing factors (attitudes, experiences, and perceived barriers towards screening).

A central question focused on whether or not HIV testing was performed, making it possible to link psychosocial determinants to observed behavior.

#### Questionnaire Validity and Reliability

The survey utilizes a theoretically validated framework, which was adapted from established public health instruments rooted deeply in the validated PRECEDE-PROCEED model [15].

To ensure rigor, we established validity and reliability through a multi-step process:

- Content and Face Validity: The conceptual boundaries of the items were governed strictly by the PRECEDE framework, drawing from prior validated studies targeting similar health behaviors. This guarantees that the predisposing, enabling, and reinforcing domains are accurately mapped.
- Expert Review: The adapted items were reviewed by a panel of health professionals and experts to ensure clarity and cultural appropriateness.
- Internal Consistency Reliability: To validate the performance of the instrument on our specific study population, we calculated Cronbach’s alpha coefficients for the main constructs. The values were about 0.70 to 0.75 for enabling factors, reinforcing factors, and for predisposing factors, demonstrating strong internal reliability (\(>0.70\)).
- To evaluate the internal consistency of the categorical binary items (Yes/No), the alpha model automatically executes the Kuder-Richardson 20 (KR-20) formula. The analysis yielded a KR-20 coefficient of 0.68 for the binary items, confirming acceptable scale reliability for our study population.

Finally, the questionnaire was administered anonymously, using accessible language (Moroccan Darija) to ensure a clear and common understanding of all items. The questionnaire was practical and easy to understand, and did not take more than 15 min to be administered.

### 2.4. Data Processing and Analysis

#### 2.4.1. Data Processing

Data was checked and coded before entry, without any missing registration. Data was exported to IBM’s Statistical Package for the Social Sciences (SPSS) version 21 software for data analysis. SPSS is software used for statistical analysis, data management, and data documentation. Descriptive statistics of data were essentially about mean, mode, and standard deviation (SD) for numerical variables and headcounts and percentages for categorical variables. A Kolmogorov–Smirnov test was performed to study the distribution of data, which reveals that the data was normally distributed with 2.148 mean and 0.38 SD. The univariate analysis used a one-sided chi-square test to recognize associations between the independent and dependent variables.

#### 2.4.2. Data Analysis

As the dependent variable was a categorical one with dichotomous choice answers, the factors identified in the univariate analysis with a *p*-value under 0.05 (*p* < 0.05) were used within a multivariate binary logistic regression model to determine potential factors influencing HIV screening adherence. The Odds Ratio coefficient and its 95% confidence interval were used to estimate the associations. The confounding issue was addressed by using a multivariate regression model. The adjusted odds ratios were directly reported to avoid ambiguity or unreadability of the model. The statistical evaluation of the model utilized the Variance Inflation Factor (VIF) for multicollinearity, which has revealed moderate and acceptable multicollinearity with a VIF of about 3.803 to 3.95 and tolerance of about 0.178 to 0.26 (VIF < 5 and tolerance > 0.1). Adjustment was done by excluding a reference category for qualitative variables. The Cox & Snell R^2^ was 0.131, and the Nagelkerke R^2^ was 0.687, which means the model explains a significant portion of the variance. Taken together, these results attest to the methodological robustness and analytical relevance of the proposed model.

The variables’ previous knowledge and test proposal were coded, such that a value of 0 corresponds to the prior knowledge or the proposition of the test, respectively, while a value of 1 corresponds to the absence of both previous knowledge and proposal of HIV screening. This methodological choice resulted in reversing the sign of the regression coefficient and producing an odds ratio less than 1, whereas the association is positive. Thus, the statistical result must be interpreted with this coding in mind: the negative coefficient reflects the chosen reference category.

### 2.5. Ethical Considerations

A data collection authorization was obtained from the regional health directorate (number: 2779\2024), and for the ethics committee, the study protocol was submitted to the University Hospital Ethic Committee (Code: 203\2024) as prescribed in common procedures. Anonymity, voluntariness, confidentiality, and written informed consent were obtained from all pregnant women participating in the study.

## 3. Results

### 3.1. HIV Screening Adherence by Predisposing, Enabling, and Reinforcing Factors

All 250 participants in the study fully responded to our questionnaire (100% response rate). The descriptive statistics, [mean, mode, SD, and variance for numeric variables; headcount and percentage for categorical variables] revealed that the majority of participants (47.2%) were aged between 25 and 40 years, were from urban areas (57.6%), and were married (91.6%). For the economic characteristics, 80.8% of participants declared having no formal work, and 54% had a low educational level (Table 1).

More than half of the population studied (56.8%) were in their first or second pregnancy, 42.4% of them were in their third trimester of pregnancy, and 90% of them were regularly following up on their pregnancy. For healthcare service accessibility, 74.8% of the participants were monitoring their pregnancy in public facilities, which guarantees economic accessibility, 38% of them have had more than four follow-up visits, and 45.2% declared not having received a proposition to undergo HIV screening. Regarding the reinforcing factors, 66.4% of the participants declared having no previous knowledge about HIV screening, 22.4% of them declared not having an HIV test because of a lack of information, and 48.4% of them considered HIV screening to be important for mother protection (Table 1).

The univariate data analysis revealed that gestational age (*p* = 0.05), pregnancy regular monitoring (*p* = 0.01), pregnancy monitoring facility (*p* = 0.017), previous knowledge about HIV screening (*p* = 0.001), and the proposition of HIV testing (0.001) were significantly associated with HIV screening (Table 1).

### 3.2. Factors Associated with HIV Screening Adherence According to Binary Logistic Regression

According to the binary logistic regression model, HIV screening adherence among pregnant women was positively associated with previous knowledge about HIV screening. Indeed, pregnant women without previous knowledge of HIV screening were less likely to adhere to HIV screening [OR: 0.034; 95% CI [0.013–0.094]] (the reference group for the regression was the women with previous knowledge). Additionally, the proposition of HIV testing was positively associated with HIV screening adherence, as the women reporting no HIV screening proposition were less likely to adhere to HIV screening [OR: 0.095; 95% CI [0.037–0.244]] (the reference group for the regression was the women reporting no HIV screening proposition) (Table 2).

## 4. Discussion

The results obtained through this study highlighted contrasting realities surrounding HIV testing during pregnancy in the Beni-Mellal region. Although international recommendations, particularly those of the World Health Organization (WHO), advocate for the systematic integration of testing into prenatal care, our research reveals a significant gap in the systematic proposal of the test, along with shortcomings in women’s knowledge and practice regarding HIV screening.

The studied population was predominantly aged between 26 and 40 years, married, living in urban areas, and with a low level of education. This configuration is comparable to that observed in a study conducted in Marrakech [16]. However, the prevalence of pregnant women without formal work (80.8%) in the current study suggests less exposure to formal medical information sources compared to working women, which may partially explain the low level of knowledge about the test.

Indeed, 66.4% of the women surveyed reported never having heard of HIV screening, an alarming rate that exceeds the one reported in a study conducted at Tripoli [17] where the rate was 47.9%. This lack of awareness could be explained by a lack of local educational initiatives. By way of comparison, in Côte d’Ivoire, a study showed that the inclusion of prenatal education sessions had increased the knowledge rate by 62% [18].

The women’s virtual acceptance of the test, in case they were offered the test properly and with structured counseling, was about 89.5%. This acceptance, although passive, confirms the findings of Sow et al. [19] in Dakar, where the majority of women accepted the test when it was properly explained. However, in the current study, only 54.8% of participants reported being offered the test, indicating a failure of the healthcare system to make it systematic. This lack of systematic proposition of the screening could be explained by an internal fear and stigma among professionals towards suggesting an HIV test. In fact, health workers are required to adopt a comprehensive strategy that must carefully navigate between two fundamental imperatives: the need for epidemiological surveillance to control the epidemic (public health) and the absolute protection of patient confidentiality and autonomy (right to privacy). The Moroccan health system has clearly opted for an approach that prioritizes strict professional confidentiality, autonomy and anonymity for PLHIV. This fundamental orientation aims to actively encourage testing and access to treatment. Nevertheless, the specific HIV screening consent procedures may constitute an obstacle to systematic testing offered by health providers. In Morocco, the consistently low rate of screening in pregnant women and the burden of vertical transmission raise the question about how to ensure sufficient prevention without offending the autonomy and privacy of pregnant women.

Furthermore, the gap between the positive perception of screening and its low rate of actual implementation (only 43.2% actually took the test) among pregnant women highlights a worrying paradox. Despite a general awareness of the importance of the test, particularly for the protection of the mother and newborn, there remains a gap between this awareness and concrete action. The rate of HIV screening adherence among pregnant women found in our study is supported by regional reports, where it does not exceed 43.2% in 2024. Nationally, the total number of pregnant women tested in 2021 was about 95,808. [20]. Hence, HIV screening still needs to be worked on to fulfill the gaps observed. This could be attributed, in part, to a lack of systematic protocols within certain primary care facilities, particularly in semi-urban and rural areas such as Beni-Mellal. This discrepancy also reflects a disconnection between intention and action, a phenomenon well documented in the public health literature. It highlights the need for an integrated approach that combines information, accessibility, training, and psychological support. A study conducted in the Democratic Republic of Congo revealed similar shortcomings, with less than 50% of pregnant women being screened, despite a positive perception of its usefulness [21].

Regarding the perception of the testing experience, a majority of women who took the test described it as neutral (74%), with a minority perceiving it positively. This finding highlights the importance of psychological support and the quality of communication when announcing and performing the test.

Furthermore, the analysis of the given motives by pregnant women for not undergoing the test suggests a lack of commitment to therapeutic communication, as 39.4% declare not having sufficient information about the test. This stance may reflect a lack of educational tools or time to engage in suitable educational sessions. A study conducted in Abidjan showed that the availability of visual tools and awareness-raising materials greatly helped to overcome reluctance [22].

Stigma surrounding HIV also remains an indirect barrier. Indeed, 29.6% of pregnant women mentioned the fear of the screening result and stigma as a reason for not undergoing the test. In many cultures, HIV is still perceived as a shameful disease, associated with moral judgments, which discourages both patients and caregivers from openly addressing the issue. A qualitative survey in Indonesia highlighted this phenomenon, where even healthcare professionals were reluctant to insist on screening for fear of offending patients’ feelings [23].

The results of our survey indicate that only 54.8% of women were offered this test, and 43.2% actually took it. This rate remains well below the minimum coverage requirements recommended globally. This finding was supported by other studies where the HIV testing prevalence is still beneath fixed goals, 33.7% in a study conducted in Indonesia and 45% in a study conducted in Ethiopia [23,24]. A much higher prevalence rate of HIV screening was observed in a study conducted in Uganda (85%) [25]. This large disparity could be partly explained by the differences in the national prevalence of HIV. Indeed, the low prevalence of HIV in Morocco (0.08) may fuel negative attitudes about HIV prevention and screening, both in pregnant women and health workers.

Although 89.5% declared significant acceptability of the test if it was offered properly, a significant lack of awareness about screening remains alarming. In fact, more than 66.4% of participants had never heard of HIV testing before or during pregnancy, revealing a major gap in health education, particularly in semi-urban and rural areas. This lack of awareness is a direct obstacle to achieving the first goal of the Joint United Nations Program on HIV/AIDS (UNAIDS) global “95-95-95” program (95% of people living with HIV should know their HIV status).

The analysis of the associated factors with pregnant women’s adherence to HIV screening reveals a statistically significant association between this adherence and previous knowledge about the HIV screening and also with the HIV screening proposition. Indeed, the women in our sample that have no previous knowledge about HIV screening and those to whom the test was not offered were less likely to adhere to HIV screening than the reference group (pregnant women with previous knowledge about HIV screening and those having received an HIV screening proposal). Consequently, actions should be taken to systematically offer HIV screening to all pregnant women at their first prenatal monitoring, with appropriate information and an approach based on informed consent. Several studies support the findings of our study. Indeed, previous knowledge about HIV screening is reported recurrently in the literature to have a significant impact on test acceptance, while the absence of test kits and human resources constraints are perceived as potential barriers [26,27].

To sum up, while there is clearly a willingness to adhere to the recommendations among women and professionals, the effective achievement of the WHO recommendations and the 95-95-95 targets remains a work in progress. A multisectoral approach, combining targeted training, strengthened health communication, and effective availability of screening services, is proving to be essential to bridge this gap and ensure that all pregnant women have equitable and informed access to HIV testing, which is the cornerstone of the fight against mother-to-child transmission.

This study highlighted an implicit willingness to adhere to HIV testing during pregnancy, which is hampered by a tangle of structural and communication barriers. This study was part of a body of studies conducted in the region to understand HIV screening adherence, antiretroviral treatment adherence and quality of life among PLHIV [28,29,30]. A recurring shortcoming highlighted by all these studies is mainly the lack of appropriate knowledge and sufficient educational and counseling programs. Therefore, the role of healthcare personnel, particularly midwives and nurses, appears central as a reliable channel of education and information, but remains limited without strong institutional support. On the other hand, reinforcing thematic organizations’ contribution in public awareness raising should be more effective and well structured. The results obtained should serve as a lever to adapt national strategies to local realities, with a view to equitable, effective, and sustainable maternal and neonatal health.

## 5. Strengths and Limitations

This study stands out for several notable methodological and scientific strengths.

The relatively high number of participants (250 women) gives the study a solid statistical basis while reflecting a diversity of sociodemographic profiles. In addition, the collection of data in the Beni-Mellal region, which is both urban and rural, enriches the analysis by incorporating territorial disparities that are often overlooked in national research. The study design has permitted the examination of multiple variables’ interference with HIV screening adherence at the same time. Additionally, the study design has permitted access to a large sample without being time- and money-consuming.

However, despite these strengths, certain limitations should be clearly highlighted. The difficulty in establishing the cause-and-effect relationships between HIV screening adherence, previous knowledge of testing, and the test proposal is due to the cross-sectional design. The sampling procedure, a non-probabilistic one, leads to selection bias and limits the generalizability of the results. The data, based on self-reported statements, may be subject to memory or social desirability biases, particularly when dealing with sensitive topics such as HIV.

To sum up, this study offers a relevant and contextualized contribution to the understanding of HIV screening in prenatal settings in Beni-Mellal city, but it would benefit from further exploration in the future, including a complementary qualitative methodology to explore perceptions in depth and also other studies addressing the topic from the healthworkers’ point of view.

## 6. Conclusions

The current study highlighted the many challenges hindering the systematic integration of HIV testing into prenatal care. Despite an overall favorable perception of this test among both patients and healthcare providers, several obstacles—informational, institutional, sociocultural, and logistical—continue to hinder universal access to this vital service.

The results revealed a striking paradox between the high acceptability of screening when it is clearly explained and its low rate of actual implementation, a direct consequence of a lack of systematization, insufficient training of healthcare providers, and often inadequate communication. This reality calls for a profound overhaul of the perinatal HIV prevention strategy, based on strengthening the capacities of professionals, ongoing awareness-raising among pregnant women, and close coordination between different levels of care.

The current study highlighted two essential factors associated with HIV screening adherence: previous knowledge about HIV testing and the test proposal. It therefore appears essential to develop targeted and ongoing training programs for healthcare personnel, particularly in the areas of empathetic communication and screening management, to establish a culture of systematic offer of HIV test to all pregnant women and all targeted populations for HIV screening. At the same time, it is essential to develop information materials adapted to the sociocultural context of patients and to promote community awareness campaigns aimed at raising HIV test awareness and knowledge in the general population and in specific targeted groups such as pregnant women. Raising awareness is also a way to fight the stigma associated with HIV, which is one of the perceived barriers to HIV screening.

Finally, this study highlights the urgent need for strong institutional commitment to ensure equitable access to HIV testing services, regardless of place of residence or socioeconomic status, in order to guarantee their systematic proposition to every pregnant woman. Only a comprehensive approach, incorporating training, infrastructure, awareness-raising, and psychological support, will enable prenatal HIV testing to become established as an essential, routine, and universal healthcare procedure. Protecting mothers and children against HIV transmission cannot be left to chance; it must become a public health priority, supported by concrete, sustainable, and coordinated actions.

## Figures and Tables

**Table 1:** HIV screening among pregnant women by sociodemographic, economic and clinical characteristics.

Sociodemographic Characteristics		HIV Screening Conducted		*p*-Value
		Yes 108 (43.2%)	No 142 (56.8%)	
Age ranges (years)	[18–25]	49 (45.4%)	59 (41.5%)	0.74
	[25–45]	48 (44.4%)	70 (49.3%)	
	≥45	11 (10.2%)	13 (9.2%)	
Origins	Urban	64 (59.3%)	80 (56.3%)	0.69
	Rural	44 (40.7%)	62 (43.7%)	
Marital status	With spouse	100 (92.6%)	129 (90.8%)	0.65
	Without spouse	8 (7.4%)	13 (9.2%)	
Occupation	Formal work	19 (17.6%)	29 (20.4%)	0.62
	Informal work	89 (82.4%)	113 (79.3%)	
Medical insurance	With health insurance	107 (71.8%)	53 (69.8%)	0.47
	Without health insurance	55 (28.2%)	35 (30.2%)	
Educational level	Low	62 (57.4%)	73 (51.4%)	0.37
	High	46 (42.6%)	69 (48.6%)	
Parity	[0–2]	62 (56.9%)	80 (56.3%)	0.71
	[2–4]	29 (26.9%)	40 (28.2%)	
	≥4	17 (15.7%)	22 (15.5%)	
Gestational age (Months)	[0–4]	40 (37.0%)	29 (20.4%)	0.05
	[4–7]	33 (30.6%)	42 (29.6%)	
	[7–9]	35 (32.4%)	71 (50.0%)	
Regular pregnancy monitoring	Yes	103 (95.4%)	122 (85.9%)	0.01
	No	5 (4.6%)	20 (14.1%)	
Number of pregnancy monitoring visits	[0–2]	16 (14.8%)	30 (21.1%)	0.17
	[2–4]	54 (50.0%)	55 (38.7%)	
	≥4	38 (35.2%)	57 (40.2%)	
Pregnancy monitoring health facility	Public	90 (85.7%)	97 (72.4%)	0.01
	Private	15 (14.3%)	37 (27.6%)	
Previous knowledge about HIV screening	Yes	71 (65.7%)	13 (9.2%)	0.001
	No	37 (34.3%)	129 (90.8%)	
HIV screening knowledge source	Midwife/nurse	10 (14.0%)	5 (38.5%)	0.66
	Physician	12 (15.3%)	6 (46.2%)	
	Others	49 (70.7%)	2 (15.3%)	
Proposal of HIV screening	Yes	3 (2.6%)	23 (53.5%)	0.001
	No	114 (97.4%)	20 (46.5%)	
Reasons for not undergoing HIV screening	Lack of knowledge		56 (39.4%)	0.03
	Fear of stigma		42 (29.6%)	
	Non-perception of risk		44 (31.0%)	
Perception of HIV screening importance	Mother protection	61 (56.5%)	60 (42.3%)	0.34
	Fetus protection	25 (23.1%)	45 (31.7%)	
	Transmission prevention	22 (20.4%)	37 (26.1%)	

**Table 2:** The associated factors to HIV screening among pregnant women according to a multivariate binomial logistic regression model.

Associated Factors	B	SE	Wald	*p*-Value	Exp (B)	95% CI
Regular pregnancy monitoring	−1.468	1.041	1.988	0.159	0.230	[0.030–1.773]
Pregnancy monitoring health facility	0.461	0.585	0.622	0.430	1.586	[0.504–4.987]
HIV screening previous knowledge	−3.37 3	0.513	43.255	0.001	0.034	[0.013–0.094]
HIV screening proposal	−2.357	0.482	23.898	0.001	0.095	[0.037–0.244]
Perception of HIV screening importance	−1.56	0.521	0.6899	0.765	0.856	[0.309–2.375]
Reasons for not undergoing HIV screening	0.375	7.730	0.025	1.250	1.455	[0.01–9.705]

## Data Availability

The data presented in this study are available on request from the corresponding author due to privacy of data and ethical concern.
